# Membrane fatty acid composition and fluidity are involved in the resistance to freezing of *Lactobacillus buchneri* R1102 and *Bifidobacterium longum* R0175

**DOI:** 10.1111/1751-7915.12132

**Published:** 2014-07-01

**Authors:** Séverine Louesdon, Séverine Charlot-Rougé, Raphaëlle Tourdot-Maréchal, Marielle Bouix, Catherine Béal

**Affiliations:** 1 UMR 782 Génie et Microbiologie des Procédés Alimentaires, AgroParisTech – INRAThiverval-Grignon, 78850, France; 2 Laboratoire Procédés Bactéries, Lallemand SASBlagnac Cedex, 31702, France; 3 UMR 02.102 PAM, Institut Universitaire de la Vigne et du Vin ‘Jules Guyot’, Université de Bourgogne - AgrosupDijon, 21000, France

## Abstract

Determinations of membrane fatty acid composition and fluidity were used together with acidification activity and viability measurements to characterize the physiological state after freezing of *Lactobacillus buchneri* R1102 and *Bifidobacterium longum* R0175 cells harvested in the exponential and stationary growth phases. For both strains, lower membrane fluidity was achieved in cells harvested in the stationary growth phase. This change was linked to a lower unsaturated-to-saturated fatty acid ratio for both strains and a higher cyclic-to-saturated fatty acid ratio for *L. buchneri* R1102 alone. These membrane properties were linked to survival and to maintenance of acidification activity of the cells after freezing, which differed according to the strain and the growth phase. Survival of *B. longum* R0175 was increased by 10% in cells with low membrane fluidity and high relative saturated fatty acid contents, without any change in acidification activity. Acidification activity was more degraded (70 min) in *L. buchneri* R1102 cells displaying low membrane fluidity and high saturated and cyclic fatty acid levels. Finally, this study showed that membrane modifications induced by the growth phase differed among bacterial strains in terms of composition. By lowering membrane fluidity, these modifications could be beneficial for survival of *B. longum* R0175 during the freezing process but detrimental for maintenance of acidification activity of *L. buchneri* R1102.

## Introduction

Lactic acid bacteria and bifidobacteria are involved in a number of industrial applications as a result of their metabolic properties and probiotic functionalities (Roy, 2005; Giraffa *et al*., 2010). *Lactobacillus buchneri* is used as a starter to improve the aerobic stability of silages by acidifying the substrate and reducing growth of yeast and molds (Giraffa *et al*., 2010). *Bifidobacterium longum* displays many probiotic benefits such as a reduction of lactose malabsorption, prevention of gastrointestinal and respiratory infections, and modulation of immune response (Aureli *et al*., 2011; Shiby and Mishra, 2013). Industrial starters of these bacteria are produced by fermentation and stabilized by freezing, freeze-drying or spray-drying under optimal conditions in order to achieve high concentrations of viable and active cells at the end of production and during the storage of the starters (Carvalho *et al*., 2004; Béal *et al*., 2008). However, since bifidobacteria grow in anaerobic environments and ferment glucose through the bifid shunt, instead of the glycolytic pathway used by lactic acid bacteria, due to of differences in their metabolism, their production conditions are not the same. Nevertheless, in both cases, exposure to freezing or freeze-drying affects their viability and their metabolic activity as a result of stressful conditions (Fonseca *et al*., 2001; Jayamanne and Adams, 2006). Different bacterial stress responses are observed at the proteome and membrane levels (Casadei *et al*., 2002; Cohen *et al*., 2006; Streit *et al*., 2008; Wang *et al*., 2011). It was previously demonstrated that the bacterial resistance to various stresses can be improved by exposing the cells to a moderate stress in order to activate these physiological responses before the real stress (Wouters *et al*., 2001; Streit *et al*., 2008; Hua *et al*., 2009). More specifically, harvesting time was shown to affect the resistance to drying of *L. rhamnosus* GG (Ampatzoglou *et al*., 2010), to freezing of *L. bulgaricus* (Rault *et al*., 2010) and to cold storage at 7°C of *L. acidophilus* (Brashears and Gilliland, 1995). However, the adaptation of *L. buchneri* and *B. longum* to freeze-drying via the modification of culture conditions has never been studied. In this context, this study aims at better understanding some of the mechanisms that govern the cryotolerance of two strains, *L. buchneri* R1102 and *B. longum* R1075 by linking their viability and acidification activity to their membrane characteristics, after harvesting cells in the exponential and stationary phases.

## Results and discussion

### Effect of harvesting time on membrane fatty acid composition and fluidity

The relative percentages of the membrane fatty acids of *L. buchneri* R1102 and *B. longum* R0175 were determined by considering cells recovered in exponential and stationary phases (Table 1). A total of eight and seven different fatty acids, representing more than 99% of the total fatty acid contents, were observed in the membranes of *L. buchneri* R1102 and *B. longum* R0175, respectively, regardless of the experimental conditions. *Lactobacillus buchneri* R1102 contained four main fatty acids, representing 96% of total fatty acid composition: palmitic acid (C16:0), palmitoleic acid (C16:1), oleic acid (C18:1) and lactobacillic acid (cycC19:0). *Bifidobacterium longum* R0175 membranes were characterized by four main fatty acids, which accounted for 97% of the total fatty acid content: myristic acid (C14:0), palmitic acid (C16:0), stearic acid (C18:0) and oleic acid (C18:1). As seen in Table 1, significant differences in the contents of some membrane fatty acids appeared, depending on harvesting conditions. For *L. buchneri* R1102, the C12:0, C14:0, C16:1 and C18:0 levels remained unchanged (*P* > 5%). In contrast, the percentages of C16:0 and cycC19:0 were higher (*P* < 0.1%), whereas the C18:1 and C18:2 relative contents were lower (*P* < 0.1%) in cells recovered in the stationary phase compared with those recovered in the exponential phase. These results suggested that C18:1 was converted into cycC19:0, as previously observed in *Pediococcus* sp (Annous *et al*., 1999). For *B. longum* R0175, the relative concentrations in C12:0 and C16:1 did not significantly change (*P* > 5%). However, stationary phase cells displayed higher relative contents of C14:0 (*P* < 0.1%), C16:0 (*P* < 0.1%) and cycC19:0 (*P* < 1%) as a result of lower percentages of C18:0 (*P* < 1%) and C18:1 (*P* < 0.1%) compared with exponential phase cells.

**Table 1:** Relative membrane fatty acid composition of *L. buchneri* R1102 and *B. longum* R0175, harvested in the exponential (H1) and stationary phases (H2)

Fatty acids (%)	*L. buchneri* R1102		*B. longum* R0175	
	H1	H2	H1	H2
C12:0	0.4 ± 0.2^a^	0.4 ± 0.1^a^	0.7 ± 0.1^a^	0.7 ± 0.1^a^
C14:0	2.2 ± 0.2^a^	2.3 ± 0.3^a^	8.7 ± 2.3^a^	20.3 ± 8.4^b^
C16:0	46.3 ± 1.7^a^	51.2 ± 2.0^b^	52.8 ± 2.7^a^	62.5 ± 5.6^b^
C16:1	8.7 ± 0.6^a^	9.2 ± 0.8^a^	1.7 ± 0.1^a^	1.8 ± 0.1^a^
C18:0	0.5 ± 0.1^a^	0.4 ± 0.1^a^	8.2 ± 1.7^b^	5.6 ± 1.0^a^
C18:1	30.7 ± 1.5^b^	14.2 ± 2.0^a^	27.2 ± 3.6^b^	7.9 ± 2.6^a^
C18:2	0.3 ± 0.1^b^	0.1 ± 0.1^a^	0^a^	0^a^
cycC19:0	10.2 ± 1.7^a^	21.3 ± 4.1^b^	0.6 ± 0.2^a^	1.1 ± 0.6^b^
Total (%)	99.3	99.1	99.9	99.9
U/S	0.80^b^	0.43^a^	0.42^b^	0.11^a^
C/S	0.21^a^	0.39^b^	0.01^a^	0.01^a^

Values are means of four measurements ± standard deviation.

Multiple comparison tests were carried out for each strain independently. Superscript letters show difference at *P* < 0.05.

These results led to the calculation of the ratios between unsaturated and saturated fatty acids (U/S), and between cyclic and saturated fatty acids (C/S) (Table 1). For both strains, the U/S ratio was higher when the cells were harvested in the exponential phase compared with the stationary phase (× 1.9 for *L. buchneri* R1102 and × 3.7 for *B. longum* R0175). The C/S was increased (× 1.9) in stationary-phase cells of *L. buchneri* R1102. These results are in agreement with those obtained with *Oenococcus oeni* (Drici-Cachon *et al*., 1996) and *Escherichia coli* (Casadei *et al*., 2002), as similar changes were found in cellular fatty acid composition according to the growth phase. In contrast, results obtained with *B. longum* R0175 partly diverged because the C/S ratio remained unchanged between the two harvesting times.

In order to investigate the possible influence of these modifications on membrane fluidity, anisotropy measurements were performed on cells harvested in exponential and stationary phases. Measurements were first carried out at 37°C to determine the initial anisotropy (r_i_) of cellular suspensions. As illustrated in Table 2, r_i_ differed according to the strain considered. *Lactobacillus buchneri* R1102 depicted significantly lower r_i_ values and, thus, higher membrane fluidity than *B. longum* R0175, for a given harvesting time (*P* < 5%). This difference could be partly explained by the higher relative saturated fatty acid content in *B. longum* R0175 compared with *L. buchneri* R1102. In addition, for both strains, cells harvested in the stationary phase showed higher r_i_, i.e., lower fluidity than cells recovered in the exponential phase (*P* < 1%). These results are in accordance with those of Cao-Hoang and colleagues (2008), which exhibited lower membrane fluidity in stationary phase cells than in exponential phase cells for *E. coli* and *B. subtilis*. Changes in membrane fluidity were also observed as a consequence of cold, acid or ethanol shocks in *O. oeni* (Chu-Ky *et al*., 2005). On the basis of these results, *L. buchneri* R1102 and *B. longum* R0175 cells were able to modulate their membrane fluidity through modifications in fatty acid composition by saturation, isomerization, cyclization, branching and acyl chain length modification, as previously suggested (Denich *et al*., 2003; Mykytczuk *et al*., 2007; Zhang and Rock, 2008).

**Table 2:** Changes in fluorescence anisotropy of *L. buchneri* R1102 and *B. longum* R0175 cells harvested in the exponential (H1) and stationary phases (H2) before and after cooling at 2000°C min^−1^ from 37 to 0°C, followed by re-heating to 37°C at 20°C min^−1^

Anisotropy	*L. buchneri* R1102		*B. longum* R0175	
	H1	H2	H1	H2
r_i_	0.136 ± 0.003^a^	0.144 ± 0.004^b^	0.148 ± 0.009^a^	0.178 ± 0.010^b^
r_m_	0.258 ± 0.011^a^	0.276 ± 0.001^b^	0.271 ± 0.006^a^	0.281 ± 0.014^a^
r_f_	0.150 ± 0.004^a^	0.159 ± 0.004^a^	0.155 ± 0.004^a^	0.176 ± 0.012^b^

Standard deviations were calculated from two independent experiments with six measurements for each experiment. Multiple comparison tests were carried out for each strain independently. Superscript letters show difference at *P* < 0.05.

In order to determine the ability of the cells to recover or not their membrane fluidity after a stress, anisotropy was measured before (r_i_), after a cold shock (from 37 to 0°C at 2000°C min^−1^, r_m_) and after re-heating (from 0 to 37°C at 20°C min^−1^, r_f_) the cell suspensions. When the cold shock was applied during anisotropy measurements, an increase of anisotropy (+163% to +194%) was observed for both strains and harvesting times, thus corresponding to a membrane rigidification (Table 2). By considering the confidence intervals, the gaps between initial (r_i_) and maximal (r_m_) anisotropy were not significantly different for *L. buchneri* R1102 cells recovered in exponential (0.122 ± 0.02) and stationary (0.132 ± 0.01) phases. In contrast, the difference between r_i_ and r_m_ was significantly lower for *B. longum* R0175 cells harvested in stationary phase cells (0.103 ± 0.05) compared with exponential phase cells (0.123 ± 0.02), thus indicating that *B. longum* was able to maintain higher fluidity during cooling when the cells were picked up in the exponential phase. After re-heating the cell suspensions, anisotropy decreased until the final anisotropy value (r_f_). This reduction indicated that rigidification was reversible for *B. longum* R0175 as r_f_ values were similar to r_i_ values for both exponential and stationary phase cells. In contrast, stationary and exponential phase cells of *L. buchneri* R1102 did not return to their r_i_ level as final values were 10% higher than initial ones. These results partly differed from those obtained by Cao-Hoang and colleagues (2008), who displayed a permanent rigidification of exponential-phase cells of *E. coli* and *B. subtilis* after rapid cooling from 37 to 0°C and re-heating at 37°C compared with stationary phase cells. Our results also revealed that bacterial membranes of *B. longum* R0175 were less affected by the cold shock than those of *L. buchneri* R1102. This discrepancy suggests that regulation of membrane fluidity depended on the bacterial genus and species, as previously observed by Cao-Hoang and colleagues (2008). This could be explained by the different fatty acid membrane compositions that characterized the membranes of the two strains (Table 1). Finally, our results revealed that the membrane of *L. buchneri* R1102 was more affected by the cold shock, thus indicating that this stress had a significant impact on the membrane integrity of these bacteria compared with *B. longum* R0175, which was less disturbed.

### Effect of harvesting time on cell survival and acidification activity after freezing

**Figure 1:**
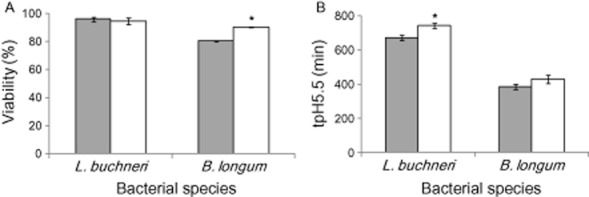
Viability (A) and acidification activity (B) of *L. buchneri* R1102 and *B. longum* R0175 harvested in the exponential (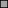) and stationary phases (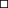) after freezing. tpH5.5, time necessary to reach pH5.5 (in minutes). Statistically significant differences (*P* < 0.05) were determined by Newman–Keuls tests and are indicated with asterisks.

In order to assess the effect of harvesting time on the survival of *L. buchneri* R1102 and *B. longum* R0175 after freezing, percentages of viable cells were quantified by double-staining with propidium iodide (PI) and carboxyfluorescein diacetate (cFDA), associated with flow cytometry detection (Fig. 1). As seen on Fig. 1A, the viability of *L. buchneri* R1102 after freezing was not significantly influenced by harvesting time (*P* > 5%). This result was consistent with other observations (Brashears and Gilliland, 1995) that demonstrated no difference in the cell survival rate between log phase cells and stationary phase cells for three *L. acidophilus* strains after freezing. In contrast, another study reported higher survival for cells of *L. acidophilus* CRL639 recovered in the stationary phase (Lorca and Font de Valdez, 1999). By considering *B. longum* R0175, significantly higher survival (*P* < 1%) was obtained when the cells were harvested in the stationary phase (90%) rather than in the exponential phase (80%). These values were correlated to similar percentages of dead cells and altered cells within the two conditions. Although the difference was only 10%, it was considered as significant. Our results differed from those of Saarela and colleagues (2005) who indicated that fermentation time did not affect the survival of freeze-dried *B. animalis* ssp. *lactis* E-012010. This difference could be explained by the lower intrinsic resistance of *B. longum* to various stresses compared with other *Bifidobacterium* species (Simpson *et al*., 2005).

The resistance of the two strains to freezing was also characterized on the basis of acidification activity measurements (Fig. 1B). The best acidification activity that corresponds to the lower time to reach pH 5.5, was achieved when *L. buchneri* R1102 cells were harvested in the exponential phase compared with the stationary phase (*P* < 1%). On the contrary, by taking the standard deviations into account, cells of *B. longum* R0175 demonstrated similar acidification activity, regardless of the harvesting time (*P* > 5%). Such differences in the resistance of the cells according to their acidification activity were previously observed with *L. bulgaricus* (Rault *et al*., 2009) and *L. acidophilus* (Lorca and Font de Valdez, 1999; Wang *et al*., 2011). This diversity could be attributed to changes that occur both at the proteome and membrane levels between the exponential and stationary phases (Casadei *et al*., 2002; Koistinen *et al*., 2007).

### Relationships between membrane properties and resistance to freezing as a function of harvesting time

**Figure 2:**
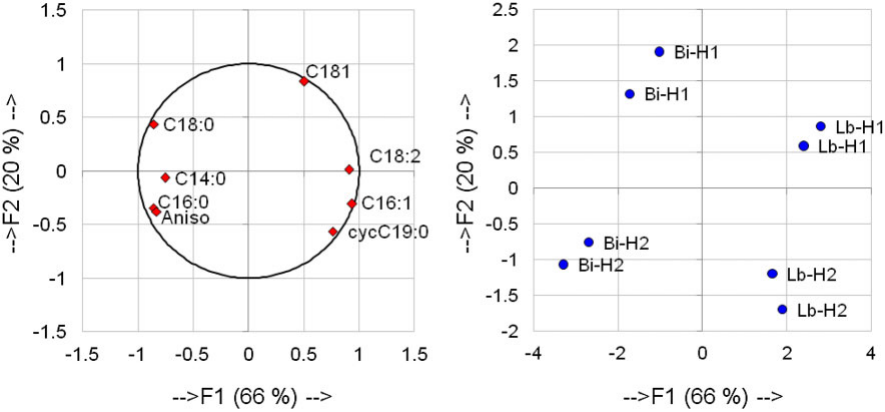
Principal component analysis linking membrane fatty acid composition and anisotropy of *L. buchneri* R1102 (Lb) and *B. longum* R0175 (Bi), harvested in the exponential (H1) and stationary phases (H2).

Principal component analysis (PCA) was performed to link membrane fatty acid composition and fluidity to harvesting time and strain specificity (Fig. 2). The following variables were retained: C14:0, C16:0, C16:1, C18:0, C18:1, C18:2, cycC19:0 and initial anisotropy. The first two dimensions of the PCA accounted for 86% of the data variance. The F1 axis (66% of the total variation) made it possible to differentiate the starters into two groups, according to their strain specificity. The group that included *L. buchneri* R1102 cells was located on the right side of the graph, whereas the group corresponding to *B. longum* R0175 cells was located on the left side of the graph. On the basis of this analysis, it could be established that *B. longum* R0175 cells were differentiated from *L. buchneri* R1102 cells by higher relative concentrations of C14:0, C16:0 and C18:0, and lower membrane fluidity, whereas *L. buchneri* R1102 starters had higher relative concentrations of C16:1, C18:1, C18:2 and cycC19:0 compared with *B. longum* R0175. The F2 axis (20% of the total variation) made it possible to distinguish the starters into two groups according to the harvesting time. The first group characterized cells harvested in the exponential phase (H1) and was located in the upper part of the graph. The second group, situated in the lower part of the graph, was composed of starters harvested in the stationary phase (H2). Stationary phase cells were differentiated from exponential phase cells by lower relative concentrations of C18:1 and higher percentages of cycC19:0 for both strains and, to a lesser extent, by higher membrane anisotropy and C16:0 content and lower C18:0 levels. As illustrated in Fig. 2, the two fatty acids, C14:0 and C18:2, did not allow differentiation of the cells according to harvesting time.

On the basis of these results, it could be established that exponential phase cells displayed higher fluidity than stationary phase cells, which was expected as membrane fluidity is essential for cell division in the exponential phase (Castuma *et al*., 1993). The higher rigidity of stationary phase cells could be explained by a combination of two stress factors, the accumulation of lactic acid in the medium that generates acid stress (Chu-Ky *et al*., 2005) and the reducing conditions that are reached at the end of the culture (Ouvry *et al*., 2002). Moreover, as shown in Fig. 2, changes in membrane fluidity were partly related to membrane fatty acid composition. Low anisotropy, i.e., high membrane fluidity, was linked to high relative contents of C18:1 but low percentages of C16:0 for the two strains. By considering each strain separately, high membrane fluidity of *L. buchneri* R1102 was also defined by a high percentage of C18:2 and a low relative content of cycC19:0, whereas that of *B. longum* R0175 was increased in membranes containing a low relative concentration of C14:0 but a high percentage of C18:0. These results indicated that the two strains used similar strategies to modulate their fluidity in the stationary phase, with some differences attributed to strain specificity. Even if both strains decreased the unsaturated fatty acid content and increased the C16:0 concentrations in their membranes to reduce their fluidity, *L. buchneri* R1102 also enhanced the fatty acid content of cycC19:0, whereas *B. longum* R0175 increased the relative level of C14:0. Consequently, depending on the strain considered, either cyclization or saturation was performed to decrease membrane fluidity as a result of the stationary growth phase.

Higher cell survival after freezing was obtained for stationary phase cells of *B. longum* R0175. On the basis of our previous results, it could be established that this behaviour was related to low unsaturated fatty acid content and a low U/S ratio as a result of the decrease in the relative content of C18:1 and the increase in the relative contents of C14:0 and C16:0. For *L. buchneri* R1102, better recovery of acidification after freezing was achieved when the cells were harvested in the exponential phase. This biological response was related to a high U/S ratio as a result of the decrease in C16:0 and the increase in the relative content of C18:1, and to a low C/S ratio due to a low percentage of cycC19:0. Such relationship between cryotolerance, membrane fatty acid composition and membrane fluidity is in agreement with the results described by Muller and colleagues (2011) with *L. johnsonii* NCC533 cells cultured in growth medium supplemented with unsaturated fatty acids. Moreover, by acting on membrane fluidity, these changes at the membrane level could be involved in the regulation and activity of membrane proteins (Beney and Gervais, 2001).

Finally, these relationships demonstrated that survival and maintenance of acidification activity in *L. buchneri* R1102 and *B. longum* R0175 were triggered by specific modifications of membrane composition that are strain- or species-dependent, leading to changes in membrane fluidity. In addition to these observations, modifications at the proteome level may also occur, as previously shown (Cohen *et al*., 2006; Koistinen *et al*., 2007; Wang *et al*., 2011). Taking these physiological changes into account, the viability of *B. longum* was improved by harvesting the cells in the stationary phase, whereas acidification activity was not modified. Even if the cell viability of *L. buchneri* did not change with harvesting time, acidification activity was better maintained in cells recovered in the exponential phase. Other strategies can be implemented to increase cell viability during starter production, either by modifying fermentation conditions (Siaterlis *et al*., 2009; Zhang *et al*., 2010; Wang *et al*., 2011), by adding specific fatty acids in the culture medium (Muller *et al*., 2011) or by submitting cells to a moderate stress (Koch *et al*., 2007; Streit *et al*., 2007; Saarela *et al*., 2009).

## Experimental procedures

### Bacterial strains and media

Frozen aliquots of *L. buchneri* R1102 and *B. longum* R0175 (Lallemand SAS, Blagnac, France) were stored at −80°C in de Man, Rogosa and Sharpe (MRS) broth (Biokar Diagnostics, Beauvais, France) supplemented with glycerol (28%). Before inoculation in the bioreactor, cells were subcultured twice for 24 h at 37°C in MRS broth.

### Fermentation and cooling

For starter production, the culture media were composed of MRS with the addition of 50 g l^−1^ glucose for *L. buchneri* R1102, and 15 g l^−1^ glucose and 20 g l^−1^ lactose (Merck, Darmstadt, Germany) for *B. longum* R0175. They were prepared and sterilized in 2.2 l fermentors (either LSL Biolafitte, Saint-Germain-en-Laye, France, or BioFlo 110, NBS, Talmadge, NJ, USA) at 115°C for 15 min. Inoculation was done at an initial concentration of 2 × 10^7^ CFU ml^−1^ for *L. buchneri* R1102 and 4 × 10^6^ CFU ml^−1^ for *B. longum* R0175. Cultivability (in cfu ml^−1^) was measured in triplicate by plate counts on MRS agar (AES-Chemunex, Combourg, France). The plates were incubated for 48 h at 37°C under aerobiosis for *L. buchneri* R1102 and anaerobic conditions for *B. longum* R0175. For both strains, fermentations were performed in duplicate at 37°C, 120 r.p.m. and pH 6.2, by adding 16% NH_4_OH solution. Fermentors were flushed with air (0.1 vvm) for *L. buchneri* R1102 and with N_2_/CO_2_ (80/20) (0.1 vvm) for *B. longum* R0175. Conductivity measurements were used to characterize fermentation kinetics and to stop the fermentations.

### Concentration and stabilization of the starters

Starters were recovered in both log and stationary phases. Harvesting of *L. buchneri* R1102 was performed when a conductivity variation of 8.3 ± 1.1 mS cm^−1^ (log phase, corresponding to 1.3 ± 0.1 10^9^ CFU ml^−1^) and 17.2 ± 0.4 mS cm^−1^ (stationary phase, 2.1 ± 0.1 10^9^ CFU ml^−1^) was achieved. For *B. longum* R0175, cultures were recovered at 11.6 ± 1.0 mS cm^−1^ (log phase, 1.8 ± 0.7 10^9^ CFU ml^−1^) and 31.9 ± 5.3 mS cm^−1^ (stationary phase, 3.7 ± 1.5 10^9^ CFU g^−1^) respectively. The cell suspensions were centrifuged (13 000 *g*, 15 min, 8°C). The supernatant was discarded, and cell pellets were frozen at −80°C before determining membrane fatty acid composition and fluidity, acidification activity, and cellular viability.

### Survival measurements

Live/dead assays were performed by dual staining each sample with cFDA and PI to differentiate viable, dead and damaged cells (Rault *et al*., 2007). Cell counts measured by flow cytometry were well correlated to plate counts (*R*^2^ = 0.946 for *B. longum* R0175 and *R*^2^ = 0.918 for *L. buchneri* R1102). Before staining, cell suspensions were diluted in McIlvaine buffer, pH 7.3 (0.2 mol l^−1^ Na_2_HPO_4_, 0.01 mol l^−1^ citric acid) (Fisher Chemical, Elancourt, France) to reach 10^6^ cells ml^−1^. One millilitre of the diluted suspension was supplemented with 10 μl of PI (Sigma Aldrich, Lyon, France) at 0.1496 mM for *B. longum* R0175 and 1.496 mM for *L. buchneri* R1102 in distilled water, plus 10 μl cFDA (0.217 μM in acetone, Invitrogen-molecular Probes, Eragny-sur-Oise, France). Samples were incubated for 10 min at 40°C before direct analysis by flow cytometry on a CyFlow® space cytometer (Partec GmbH, Münster, Germany). Viable cells were identified as simultaneously showing fluorescence with cFDA and no fluorescence with PI. Averages of two measurements were used to calculate percentages of viable cells after freezing.

### Acidification activity measurements

The Cinac system (Ysebaert, Frépillon, France) was used to measure the acidification activity of cells before and after freezing (Fonseca *et al*., 2000). Acidification was performed at 37°C, in triplicate, in Erlenmeyer flasks containing 120 ml MRS broth. Similar inoculation rates were used for both harvesting times using 0.2 ml (*B. longum* R0175) and 0.3 ml (*L. buchneri* R1102) of frozen culture, diluted 10 times. The time necessary to reach pH 5.5 (tpH5.5, in min) was used to characterize the acidification activity of the bacterial suspensions. The higher the tpH5.5 was, the longer the latency phase was, and consequently, the lower the acidification activity was.

### Fatty acid analyses

The relative fatty acid composition of bacterial membranes was determined by gas chromatography (Béal *et al*., 2001). Cell pellets were washed three times in 0.05 M Tris buffer, pH 8.8. Methylation and extraction were simultaneously performed at 4°C by adding 1.5 ml sodium methoxide (1 M in methanol, Sigma-Aldrich), shaking for 2 min and adding 1 ml hexane (Sigma-Aldrich). After the addition of undecanoic acid methyl ester (0.1 mg ml^−1^ in hexane) as internal standard and decantation for 5 min, the upper phase was removed and stored at −80°C in an airtight glass bottle until analysis. Analyses were performed on a gas chromatograph (HP 6890, Hewlett Packard, Avondale, PA, USA) equipped with a capillary column packed with 70% cyanopropyl polysilphenylene-siloxane (BPX 70, 60 m * 0.25 mm; SGE, Victoria, Australia). Helium was used as the carrier gas (1.2 ml min^−1^), and the injection volume was 2 μL. Injection was done splitless for 2 min. The oven temperature was raised from 65 to 230°C at 5°C min^−1^ and maintained for 10 min at 230°C. Injection and detection temperatures were 230°C. The fatty acid methyl esters were identified by using a mass selective detector (Agilent 5973, Hewlett Packard) at a scan rate of 273 scan s^−1^. The electron impact energy was set at 70 eV, and data were collected in the range of 30–400 atomic mass units. The mass spectra were compared with the NBS75K and WILEY 275.L data banks (Hewlett Packard). The identity of the fatty acid methyl esters was confirmed by comparing their retention times with those of known standards (BAME and FAME, Supelco, Bellefonte, PA, USA). Analyses were done in quadruplicate, and results were expressed as relative percentages of each fatty acid, which were calculated as the ratio of the surface area of the considered peak to the total area of all peaks.

### Determination of membrane fluidity

Fluorescence anisotropy was assessed to characterize the membrane fluidity of the cells by using a spectrofluorometer (Fluorolog-3, Jobin Yvon, USA) in a T format (Cao-Hoang *et al*., 2008). Cell pellets were washed three times (5000 *g*, 5 min, 25°C) in 50 mM MES (Sigma Aldrich) buffer {[2-(N-morpholino) ethanesulfonic acid]-KOH, 10 mM glucose, pH 5} and resuspended in the same buffer to reach an optical density of 0.6 at 600 nm. Fluorescence anisotropy was measured by using hydrophobic 1,6-diphenyl-1,3,5-hexatriene (DPH) (Invitrogen Molecular Probes), according to static and dynamic approaches. For static measurements, 2 ml of cell suspension were added with 4 μl of 1.5 mM DPH solution (in tetrahydrofuran, Sigma-Aldrich), and measurements were performed in the dark at 37°C, every 8 s for at least 10 min. For the dynamic method, measurements were conducted together with an instantaneous cooling (2000°C min^−1^), which was achieved by immersing 0.3 ml of concentrated cell suspension (×10) at 37°C into 2.7 ml of MES-KOH buffer (10 mM glucose, pH 5) at −3.4°C. The cooling kinetic was measured using a T thermocouple (TCSA, Dardilly, France) connected to an InstruNet acquisition card (GWI, Somerville, MA, USA). Samples were kept at 0°C for 10 min, and then re-heated to 37°C at 20°C min^−1^.

Excitation and emission wavelengths were set at 340 and 431 nm, respectively, and the anisotropy (r) was calculated according to Lakowicz (1988). This method made it possible to determine initial anisotropy from steady-state measurements (r_i_), maximal anisotropy after cooling (r_m_) and final anisotropy after reheating (r_f_). Calculated anisotropy values were the means of at least five assays.

### Statistical analyses

Results were compared by using analysis of variance with StatgraphicPlus Software v3.0 (Manugistics, Rockville, MD, USA). Multiple comparison tests were done according to Newman–Keuls tests for each strain independently. When *P* values were lower than 0.05, differences were considered as being statistically significant.

PCA (Statbox™ v6.7 Grimmersoft, Neuilly-sur-Seine, France) was carried out to explore links between the relative contents of membrane fatty acids, membrane fluidity viability and acidification activity, for both strains.

## Conclusion

Our study revealed that membrane fatty acid composition and fluidity of *L. buchneri* R1102 and *B. longum* R1075 were changed as a function of harvesting time but differently depending on the strain. Different mechanisms allowed membrane fluidity to be regulated by alteration in the relative concentrations of unsaturated (*B. longum* R1075) or cyclic (*L. buchneri* R1102) fatty acids, thus decreasing the saturated fatty acid content. In addition, these membrane properties were associated with the survival of *B. longum* R0175 and to the maintenance of the acidification activity of *L. buchneri* R1102 after freezing, which differed according to the growth phase. Finally, the fatty acid composition and the fluidity of bacterial membranes were identified as important characteristics to be considered in order to improve starter quality. In the future, these results may be confirmed by taking additional strains into account. In addition, it will be essential to characterize membrane proteins to identify complementary biological mechanisms involved in the physiological responses to freezing.

## Conflict of interest

None declared.
